# Early-Onset Vitreous and Cardiac Amyloidosis With *TTR* Trp61Leu

**DOI:** 10.1016/j.jaccas.2026.108337

**Published:** 2026-05-15

**Authors:** Emily Li, Steve L. Liao, Maria Giovanna Trivieri, Peter Gorevic, Veronica Fettig, Anuradha Lala, Alan Sheyman, Susan Shin, Noura S. Abul-Husn, Amy R. Kontorovich

**Affiliations:** a Department of Medicine, Icahn School of Medicine at Mount Sinai, New York, New York, USA; b Fuster Heart Hospital, Icahn School of Medicine at Mount Sinai, New York, New York, USA; c Biomedical Engineering and Imaging Institute, Icahn School of Medicine at Mount Sinai, New York, New York, USA; d The Cardiovascular Research Institute, Icahn School of Medicine at Mount Sinai, New York, New York, USA; e Division of Rheumatology, Department of Medicine, Icahn School of Medicine at Mount Sinai, New York, New York, USA; f Division of Pediatric Cardiology, Department of Pediatrics, Icahn School of Medicine at Mount Sinai, New York, New York, USA; g Department of Ophthalmology, The Mount Sinai Hospital and New York Eye and Ear Infirmary of Mount Sinai, New York, New York, USA; h Department of Neurology, Icahn School of Medicine at Mount Sinai, New York, New York, USA; i Division of Genomic Medicine, Department of Medicine, Icahn School of Medicine at Mount Sinai, New York, New York, USA; j The Institute for Genomic Health, Icahn School of Medicine at Mount Sinai, New York, New York, USA; k 23andMe Research Institute, Palo Alto, California, USA

**Keywords:** amyloid, genetic, transthyretin

## Abstract

The pathogenic transthyretin (*TTR*) gene variant c.182G>T, p.Trp61Leu (Trp61Leu) was previously reported once, as variant transthyretin amyloidosis (ATTRv) manifesting with vitreous and cutaneous involvement, with symptom onset at age 42. We report updated clinical progression of Trp61Leu-associated ATTRv in an patient (the proband) and her 2 daughters, who represent the first known cases of Trp61Leu cardiac amyloidosis (CA) with onset before age 40. This early-onset CA stands in stark contrast to that observed with most other ATTRv genotypes and is further highlighted by the finding that technetium pyrophosphate scans were severely abnormal before any echocardiographic abnormalities became apparent. We also report an unrelated patient with wild-type germline *TTR* who developed ATTR after receiving the proband's transplanted liver through a domino procedure. Furthermore, vitreous amyloid preceded CA in 3 of 4 cases, highlighting an important and often overlooked “red flag” feature of ATTRv not addressed with existing transthyretin silencer or stabilizing therapies.

## Visual Summary

**Figure:**
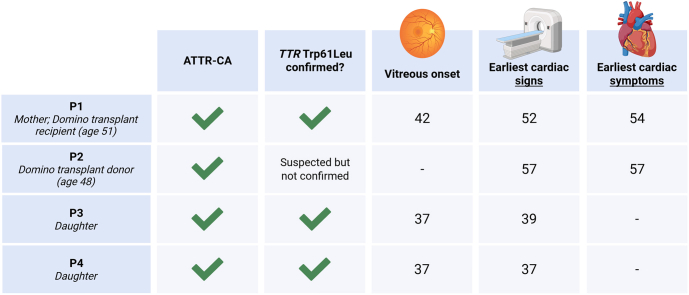

**Visual Summary:**
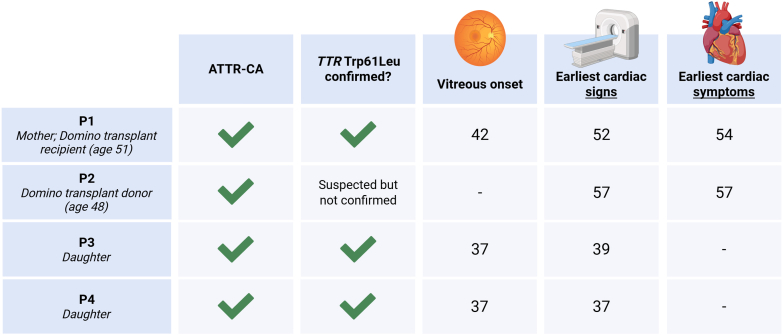
Summary of the 4 Cases With ATTR CA Reported in This Case Series Three cases were confirmed to be due to the *TTR* Trp61Leu variant and 1 (domino transplant donor) was suspected, but not confirmed, to be due to this variant. Vitreous disease was the first symptom to arise in P1 and her 2 daughters, P3 and P4 (ages of onset listed). Cardiac signs were first noted in P1 at age 52. Because of the known genotype-positive status of P3 and P4, early cardiac screening was initiated; both were confirmed on imaging to have CA more than a decade earlier than their mother's CA was first diagnosed and before the onset of any cardiac symptoms. ATTR = transthyretin amyloidosis; CA = cardiac amyloidosis; P1/2/3/4 = patient #1/2/3/4; Trp61Leu = c.182G>T, p.Trp61Leu; *TTR* = transthyretin gene.

Multiple genotype-phenotype associations are reported for the ∼130 known transthyretin (*TTR*) gene variants pathogenic for variant transthyretin amyloidosis (ATTRv). For example, the most common variant in the United States, Val142Ile, is frequently linked with later onset (age ≥60 years) cardiac amyloidosis (CA) and heart failure (HF),^1^ whereas the Val50Met variant often causes earlier-onset (beginning in the fourth decade of life) polyneuropathy (PN).^2^ In ATTRv, amyloidogenic transthyretin protein (TTR) is primarily hepatic in origin; however, TTR is also synthesized in the choroid plexus and retinal pigment epithelial cells and ophthalmic manifestations occur with 25% of *TTR* genotypes.^3^ c.182G>T, p.Trp61Leu (Trp61Leu) (previously referred to as Trp41Leu) is a rare *TTR* variant first reported in 2002 in a woman with vitreous amyloid at age 42, without evidence at that time for cardiac, neuropathic, or other systemic involvement (patient #1; the proband).^4^ Progressive peripheral and autonomic neuropathies developed later, with biopsy-proven cutaneous and bladder involvement by age 48.^5^ Here, we report updated clinical progression of Trp61Leu-associated ATTRv in the proband, her 2 daughters, and an additional unrelated patient who received a liver transplant from the proband.

**Take-Home Messages**

• Trp61Leu is a rare *TTR* variant associated with early-onset cardiac amyloidosis, occurring before age 40 in 2 newly described cases, and often preceded by vitreous amyloid, an important but under-recognized early manifestation of variant transthyretin amyloidosis.
• These cases highlight that severely abnormal technetium pyrophosphate scans may precede structural cardiac changes, underscoring the need for early nuclear scintigraphy screening in genotype-positive patients.
• Vitreous amyloidosis should be recognized as a potential “red flag” feature, prompting genetic testing, timely diagnosis, and comprehensive surveillance.

## Case Report

In the mid-2000s, at age 51, patient #1 (the proband) underwent orthotopic whole liver transplantation via a domino procedure because of progressive neuropathy. She subsequently developed chronic kidney disease and progressive autonomic dysfunction, despite treatment with diflunisal, which was the only therapeutic under investigation at the time. At age 52, echocardiography demonstrated severe concentric left ventricular (LV) hypertrophy (intraventricular septal thickness in diastole [IVSd] 17 mm; LV mass index 145 g/m^2^) with “ground glass” appearance and restrictive mitral inflow pattern and moderate right ventricular hypertrophy with decreased compliance. By age 54, the IVSd was 21 mm and LV mass index 187 g/m^2^. The patient died of end-stage HF at age 55.

Patient #2 was an Asian man with a history of transfusion-dependent thalassemia who required liver transplantation for hepatocellular carcinoma that developed in the setting of hepatitis B. At age 48, he became the recipient of patient #1's donor liver via domino transplantation. His preoperative cardiac status was stable; an echocardiogram at age 51 showed an IVSd of 10 mm. At age 57, he developed dyspnea and lower extremity edema. Repeat echocardiography revealed moderate concentric LV hypertrophy and cardiac magnetic resonance confirmed this finding (wall thickness 13-14 mm); gadolinium was not used because of the patient's advanced kidney disease. A technetium Tc-99m hydroxydiphosphonate planar whole-body scintigraphy scan reported uptake (without grading) over the heart, suggestive of amyloidosis, new in comparison to a bone scan performed before transplantation to evaluate carcinoma metastases. An initial endomyocardial biopsy showed mild hypertrophic changes but was negative for Congo red staining. However, an abdominal fat pad biopsy at age 58 stained positive for Congo red, and electromyography revealed distal axonal sensory motor PN. Repeat endomyocardial biopsy at age 60 was positive for Congo red, with liquid chromatography tandem mass spectrometry revealing transthyretin amyloidosis (ATTR). Although the Trp61Leu variant was not detected by this analysis, it could not be determined whether the cardiac ATTR was wild-type or mutant, because of the limitations of this technique, which cannot detect all sequence variations. However, his presentation occurred at a significantly younger age than has been previously reported for wild-type ATTR-CA, which typically arises in the seventh to eighth decade of life,^6^^,^^7^ supporting a possible role of the Trp61Leu variant in his disease. The patient's restrictive cardiomyopathy progressed, and he died of HF complications at age 60.

**Figure 1:**
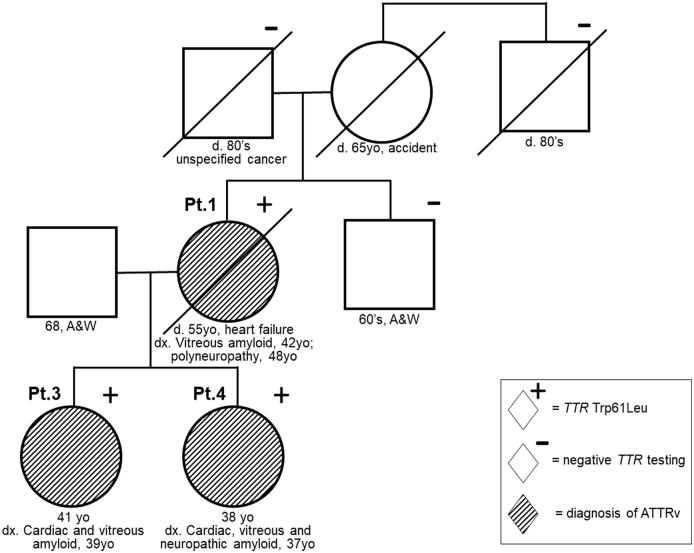
Pedigree for Related Patients in the Series Pedigree of *TTR* Trp61Leu–positive family including patient #1 (proband, black arrow) and her 2 affected daughters, patients #3 and #4. ATTRv = variant transthyretin amyloidosis; Trp61Leu = c.182G>T, p.Trp61Leu; *TTR* = transthyretin gene.

**Figure 2:**
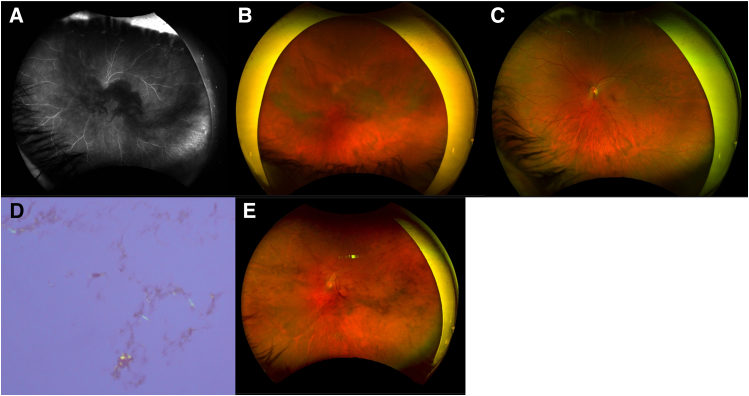
Ophthalmologic Findings in a Patient With TTR Trp61Leu (A) Fluorescein angiogram in patient #3 showing vitreous opacities in front of the retina without signs of vasculitis or optic neuritis. (B) Photograph of the fundus showing vitreous opacities in front of the retina. (C) Postsurgery, photograph of the fundus showing resolution of vitreous opacities. (D) Positive Congo red staining of the vitrectomy bag showing apple green birefringence under polarized light. (E) Photograph of the fundus 24 months postsurgery, showing recurrence of vitreous opacities.

**Figure 3:**
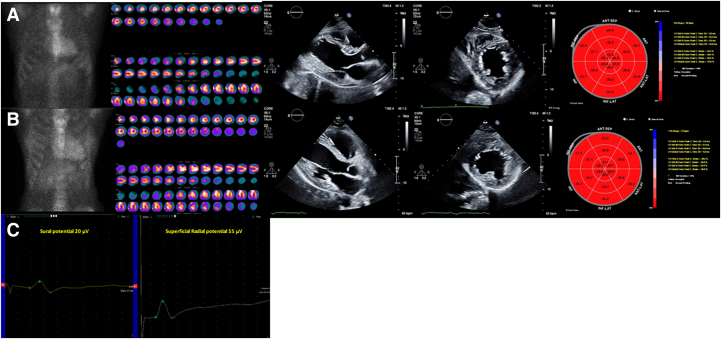
Cardiac Imaging and Nerve Conduction Findings in a Patient With TTR Trp61Leu Markedly abnormal planar (left) and cardiac (middle) single-photon emission computerized tomography images from technetium pyrophosphate studies despite normal echocardiographic findings (right) in (A) patient #3 (anteroseptal wall thickness 1.0 cm; inferolateral wall thickness 0.9 cm; global longitudinal strain −41.6%) and (B) patient #4 (anteroseptal wall thickness 0.9 cm; inferolateral wall thickness 0.9 cm; global longitudinal strain −36.6%). (C) Abnormal nerve conduction study tracings in patient #4.

Patient #3 is the eldest daughter of patient #1 (Figure 1), who developed visual floaters at age 37 and underwent left eye pars plana vitrectomy and vitreous biopsy; Congo red staining was positive (Figure 2). Genetic testing (*TTR* sequencing) revealed the familial heterozygous pathogenic *TTR* variant Trp61Leu, confirming the diagnosis of ATTRv. An echocardiogram at age 39 was overall normal, with borderline normal LV wall thickness (maximal 10 mm) and normal global longitudinal strain (−24.2%). Bone scintigraphy scanning with technetium pyrophosphate (Tc-99m PYP) including single-photon emission computerized tomography was markedly abnormal (semiquantitative score grade 3; heart-to-contralateral ratio 2.07) (Figure 3A). Serum free light chains and serum and urine immunofixation were normal. The patient was diagnosed with CA and started on tafamidis, which was the only Food and Drug Administration–approved ATTRv-CA therapy available at the time. The results of electromyography and nerve conduction testing were initially normal, but the patient endorsed intermittent paresthesias, dysphagia, and incomplete bladder voiding. Repeat testing at age 41 revealed large fiber sensory deficits in the distal lower extremities with pinprick gradient up to the shins and reduced vibration in the toes vs fingers. The sural-to-radial nerve amplitude ratio was 0.3 (normal range: ≥0.4), suggestive of early distal sensory large fiber PN, interpreted as a manifestation of ATTRv given the absence of any other neuropathy risk factors. The TTR silencer vutrisiran was added, and she continues close follow-up with the cardiovascular genetics and neurology teams.

Patient #4 is the youngest daughter of patient #1. She developed visual floaters at age 37, and genetic testing revealed the familial *TTR* Trp61Leu variant. Echocardiography showed normal LV wall thickness (maximal 9 mm) and global longitudinal strain (−23.7%). Given her sister's young age at CA onset, a Tc-99m PYP scan was performed (Figure 3B). The semiquantitative score was grade 3; the heart-to-contralateral ratio was 1.7; and serum free light chains and serum and urine immunofixation were normal. The patient had received a diagnosis of CA and started on tafamidis. Although she denied any subjective PN symptoms, on clinical examination she had reduced peripheral sensation in a stocking/glove distribution and reduced vibration sense in the toes with an abnormal sural-to-radial nerve amplitude ratio of 0.36 (Figure 3C), which, similar to her sister, was consistent with early distal sensory large fiber PN due to ATTRv. At age 37, she was also started on vutrisiran.

## Discussion

Here we describe 3 related cases with confirmed *TTR* Trp61Leu ATTR and an additional unrelated case of a patient who developed ATTR only after receipt of a transplanted liver bearing the Trp61Leu variant. To our knowledge, these are the first reports of cardiac involvement in ATTRv due to *TTR* Trp61Leu. HF due to presumed CA developed in the proband at age 52 (10 years after the initial case report was published), rapidly progressing to end-stage disease despite liver transplantation, a known phenomenon postulated because of enhanced deposition of wild-type TTR on a “template” of variant amyloid fibrils.^8^^,^^9^ In addition, in the transplantation recipient of this proband's liver, the *TTR* variant from Trp61Leu donor hepatic cells may have promoted myocardial mutant and/or accelerated wild-type TTR amyloid deposition. These 2 patients were treated in the era preceding the availability of TTR silencer and stabilizer therapies. In current practice, disease-modifying pharmacologic therapies have largely supplanted liver transplantation as the preferred treatment strategy for ATTRv. However, in the rare circumstances in which liver transplantation is considered, livers from patients harboring the Trp61Leu variant—and potentially other aggressive variants—may be unsuitable for use in domino transplantation. Care of these 2 patients also preceded the seminal 2016 study by Gillmore et al,^10^ which has since precipitated a sea change toward use of bone scintigraphy for highly sensitive and specific noninvasive diagnoses of CA. In the contemporary era, CA was diagnosed in both genotype-positive daughters under age 40 before the onset of any HF symptoms. Notably, these 2 young patients had profoundly abnormal Tc-99m PYP scans despite absence of LV hypertrophy on echocardiography. Variations in myocardial amyloid fibril composition and codeposition of dystrophic calcium have been shown to explain differences in bone tracer affinity, with lower affinities for full-length type B TTR vs type A TTR.^11^ This is believed to explain the significantly lower sensitivity of bone scintigraphy in detecting CA from certain *TTR* variants (Phe84Leu^12^ and Val50Met^13^). In contrast, amyloid fibrils generated from Trp61Leu may impart greater affinity for nuclear tracers, thereby permitting higher sensitivity for scintigraphic CA detection and enabling treatment at the earliest presymptomatic stages of disease. Further studies are needed to characterize the range of sensitivities for detection of CA via bone scintigraphy across all known *TTR* genotypes. This series underscores the clinical value of early familial cascade screening, exemplified by patients #3 and #4, who were affected but asymptomatic at the time their pathogenic genotype was identified. Cascade screening may be particularly valuable for families seeking eligibility for preventive trials such as ACT-EARLY Trial (Phase 3, Randomized, Multicenter, Double-Blind, Placebo-Controlled Study of Acoramidis for Transthyretin Amyloidosis Prevention in the Young), which is evaluating the TTR stabilizer acoramidis in younger asymptomatic carriers using a randomized controlled design.^14^ On the basis of findings in this small cohort including several related patients, we offer a preliminary recommendation for cardiac screening in Trp61Leu carriers starting at age 30 and advocate for inclusion of nuclear scintigraphy/single-photon emission computerized tomography scanning even in those without echocardiographic abnormalities or HF symptoms.

## Conclusions

In summary, *TTR* Trp61Leu is associated with early-onset CA, along with vitreous, cutaneous, bladder, autonomic, and peripheral amyloidosis. Aggressive variants such as Trp61Leu may drive early myocardial amyloid deposition detectable by PYP imaging before overt ventricular hypertrophy or HF symptoms emerge, emphasizing the critical role of early familial cascade screening through genetic testing. Because vitreous involvement may be the presenting feature for certain *TTR* genotypes, it should be included among “red flag” features for ATTRv. Finally, because the currently available TTR stabilizer and silencer therapies have not been shown to cross the blood-brain barrier, further efforts are needed toward the development of vitreous amyloidosis treatments.

## Funding Support and Author Disclosures

This work was supported by the National Institutes of Health/National Heart, Lung, and Blood Institute grant R01HL155356 and an investigator initiated award from Akcea Therapeutics. Dr Kontorovich has received research support from Pfizer. Dr Abul-Husn is an employee of the 23andMe Research Institute and a member of the clinical advisory board for Inflection Medicine. All other authors have reported that they have no relationships relevant to the contents of this paper to disclose.
